# Non-Thermal Plasma Treatment Influences Shoot Biomass, Flower Production and Nutrition of Gerbera Plants Depending on Substrate Composition and Fertigation Level

**DOI:** 10.3390/plants10040689

**Published:** 2021-04-02

**Authors:** Samantha Cannazzaro, Silvia Traversari, Sonia Cacini, Sara Di Lonardo, Catello Pane, Gianluca Burchi, Daniele Massa

**Affiliations:** 1 CREA Research Center for Vegetable and Ornamental Crops, Council for Agricultural Research and Economics, Via dei Fiori 8, 51012 Pescia, PT, Italy; samantha.cannazzaro@libero.it (S.C.); sonia.cacini@crea.gov.it (S.C.); gianluca.burchi@crea.gov.it (G.B.); daniele.massa@crea.gov.it (D.M.); 2 Research Institute on Terrestrial Ecosystems (IRET-CNR), Via Madonna del Piano 10, 50019 Sesto F.No, Florence, Italy; sara.dilonardo@cnr.it; 3 CREA Research Center for Vegetable and Ornamental Crops, Council for Agricultural Research and Economics, Via Cavalleggeri 25, 84098 Pontecagnano Faiano, SA, Italy; catello.pane@crea.gov.it

**Keywords:** bedding plants, fertilizer saving, floriculture, green compost, nitrogen, peat, reactive oxygen species, reactive nitrogen species

## Abstract

Non-thermal plasma (NTP) appears a promising strategy for supporting crop protection, increasing yield and quality, and promoting environmental safety through a decrease in chemical use. However, very few NTP applications on containerized crops are reported under operational growing conditions and in combination with eco-friendly growing media and fertigation management. In this work, NTP technology is applied to the nutrient solution used for the production of gerbera plants grown in peat or green compost, as an alternative substrate to peat, and with standard or low fertilization. NTP treatment promotes fresh leaf and flower biomass production in plants grown in peat and nutrient adsorption in those grown in both substrates, except for Fe, while decreasing dry plant matter. However, it causes a decrease in the leaf and flower biomasses of plants grown in compost, showing a substrate-dependent effect under a low fertilization regime. In general, the limitation in compost was probably caused by the high-substrate alkalinization that commonly interferes with gerbera growth. Under low fertilization, a reduction in the photosynthetic capacity further penalizes plant growth in compost. A lower level of fertilization also decreases gerbera quality, highlighting that Ca, Mg, Mn, and Fe could be reduced with respect to standard fertilization.

## 1. Introduction

The treatment of water devoted to agronomic purposes with non-thermal plasma (NTP) is receiving increasing interest following promising results obtained using this technology [1]. NTP is a weakly ionized gas that produces electrons, reactive oxygen species (ROS), reactive nitrogen species (RNS), UV radiation, and a local electric field. It is generated at air temperature using devices as dielectric barrier discharger (DBD) reactors and can be used to treat water, changing its chemical composition [2]. In particular, plasma-activated water (PAW) could present an increase in ROS and RNS and variations in the redox potential and conductivity of the water [3]. The combined effect obtained by the strong oxidant activity of ROS and water acidification, through the generation of nitric acid from RNS, confers an antimicrobial activity to PAW [4], with it thereby representing an environmentally sustainable alternative to chemical sanitizers or pesticides. In fact, plants treated with PAW show a strong inactivation of phytopathogens [5]. Moreover, plasma treatment causes the fixation of air nitrogen, increasing the concentrations of nitrite, nitrous acid, nitrate, and nitric acid, conferring fertilizing proprieties to PAW as well [2]. The use of PAW has been shown to have positive effects on plant yield. Among several examples, the use of PAW generated by DBD increases both seed germination and plant biomass in tomatoes, peppers, and radishes [6]. In fact, the benefit of low concentrations of exogenous ROS and RNS has been already shown; this is probably determined by modulation of plant metabolism and immune response [7,8]. Despite this potential role of NTP for use in agriculture, examples of PAW applications on intensive ornamental and vegetable crop cultivation are still few.

In the ornamental nursery sector, peat moss is widely used as a major component of growing media for potted plant production. However, in recent decades, the horticultural industry has been trying to reduce the use of peat as a soilless substrate due to its environmental unsustainability, related to ecological concerns [9], and its increasing price [10]. Consequently, many new plant substrates and mixtures have been introduced and tested worldwide [11]. A promising alternative growing medium, in line with circular economy concepts, consists of green compost obtained from different organic wastes. This shows potential not only in terms of its positive carbon footprint but also for its low price and suppression of some soil-borne diseases [12]. One of the main problems of compost use can be its high variability of physical and chemical characteristics [13]; however, it may have similar physical features to peat if properly selected [14]. In order to assess the suitability of an alternative substrate for potted plant production, its effects on plant yield and ornamental traits must be assessed during the entire cultivation period, especially on plants that require a specific pH range for their growing medium [15]. As an example, gerbera plants are naturally grown in acidic soils/substrates and show chlorosis symptoms and nutrient deficiency at a pH of around six [16]. The use of different peat-alternative substrates for gerbera production has been the object of several studies [17,18,19]. The main concern has been the strong influence of substrate composition on the pH and electrical conductivity (EC) of growing media, and consequently on nutrient availability [20]. As an example, the addition of coconut coir and mushroom compost lowered the pH and increased the EC of conventional substrates used for gerbera production (e.g., garden soil, silt, and sand) and consequently could improve plant nutrient uptake [21].

Ornamental potted plants are commonly maintained via protected cultivation fed with a fertigation system that provides nutrients through drip irrigation directly supplied into the active root zone, reducing nutrient release into the environment [22]. Therefore, a reduction of fertilizer concentration might not have negative effects on plants. As an example, in potted gerbera production, a reduction of up to 50% in nutrient concentration does not lead to adverse effects at the end of cultivation when using a mixture of sphagnum peat moss:perlite (4:1) as a substrate [23]. However, a 50% reduction of nutrient supply causes a decrease in growth, and flower number per plant, as well as an increase in bent neck, when using a mixture of soil:compost:sand (2:1:1) [24]. The presence of compost in growing media can indeed support the growth and development of ornamental plants by providing an extra budget of nutrients [25], possibly available to the crop, and for introducing the presence of bioactive organic compounds that may stimulate and improve plant nutrient use efficiency under fertilizer shortage [26]. Therefore, it is strongly recommended that the evaluation of fertigation strength is made in combination with the substrate composition, taking into particular consideration both pH and EC.

The aim of this work is to evaluate the effect of NTP treatment on the suitability of green compost as an alternative substrate to peat for gerbera bedding plant production, and test both substrates using a standard or a low fertilization regime, assessing the opportunity for fertilizer saving. All of these treatments and their combinations are evaluated to assess possible improvements in plant yield, plant nutrition, and ornamental traits, as well as variations in microorganism presence in the rhizosphere, when using NTP to treat the nutrient solution.

## 2. Results

### 2.1. Plant Biomass Analyses

**Figure 1:**
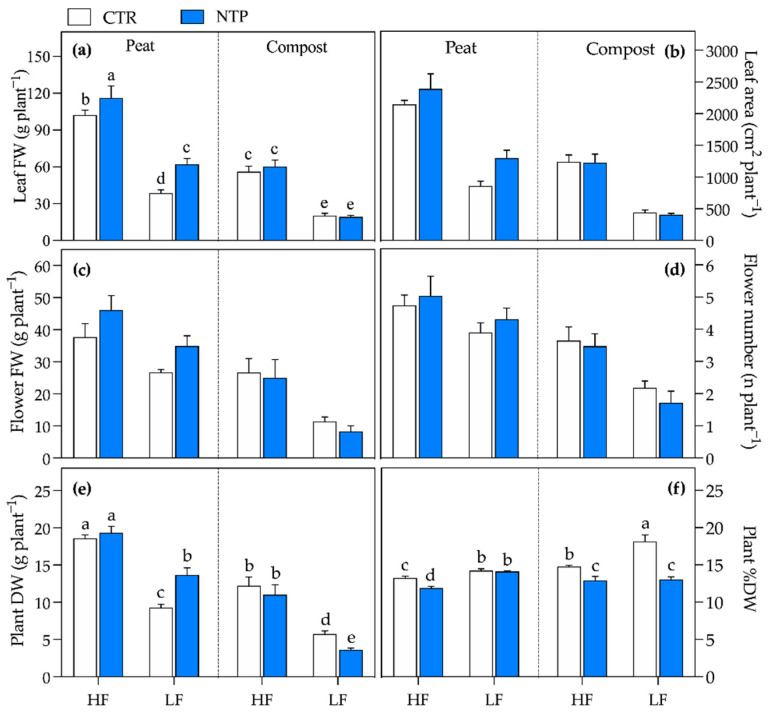
Biometric measurements of gerbera plants grown in peat or compost and fed with high (HF) or low (LF) nutrient solution, untreated (CTR) or NTP-treated (NTP): leaf fresh weight (**a**), leaf area (**b**), flower fresh weight (**c**), flower number (**d**), plant dry weight (**e**), and plant dry weight percentage (**f**). Values represent the means (*n* = 4) + SD. The presence of different letters represents Tukey’s test results for significant triple interaction, while three-way ANOVA *p*-values are reported in Table 1.

**Figure 2:**
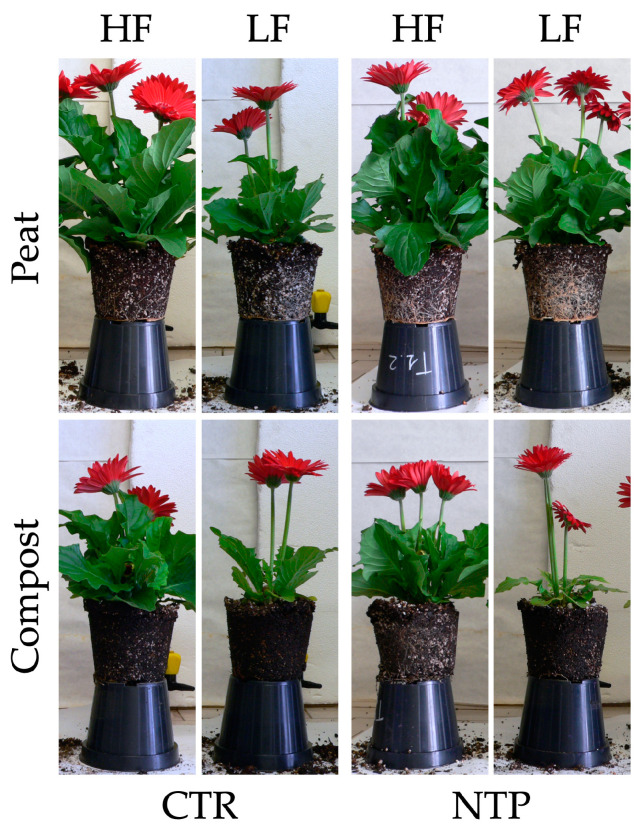
Images of gerbera plants grown in peat or compost and fed with high (HF) or low (LF) nutrient solution, untreated (CTR) or NTP-treated (NTP).

Biometric parameters of gerbera plants were simultaneously influenced by the use of NTP-treated solution, the fertilization level, and type of substrate (Figure 1, Table 1). Leaf fresh weight (FW) was higher in plants grown in peat substrates with high fertilization (HF), while the lowest value was measured in plants grown in compost under low fertilization (LF) (Figure 1a, Table 1). NTP treatment increased this parameter in peat while it did not show any effect in compost. A similar trend was measured for the leaf area (Figure 1b, Table 1). As for the leaves, the highest flower FW and number were measured under both untreated or NTP-treated conditions in peat HF plants (on average 42 ± 6.0 g plant^−1^, and 5 ± 0.5 flowers plant^−1^, respectively, Figure 1c,d and Table 1). No variation in single flower FW occurred (data not shown). Plant dry weight (DW) (Figure 1e, Table 1) ranked first in peat HF plants in both untreated and NTP-treated plants (on average 19 ± 0.8 g plant^−1^). Under an LF regime, the NTP treatment influenced the plant biomass in a substrate-dependent way, as was highlighted by a visual evaluation of plant features (Figure 2). Interestingly, NTP-treated solution increased the plant DW in peat while it decreased this parameter in compost. However, NTP did not affect plant biomass under a HF regime in the same compost-based substrate. DW concentration was higher in LF plants and compost, while plants treated with NTP showed a higher degree of tissue hydration.

**Figure 3:**
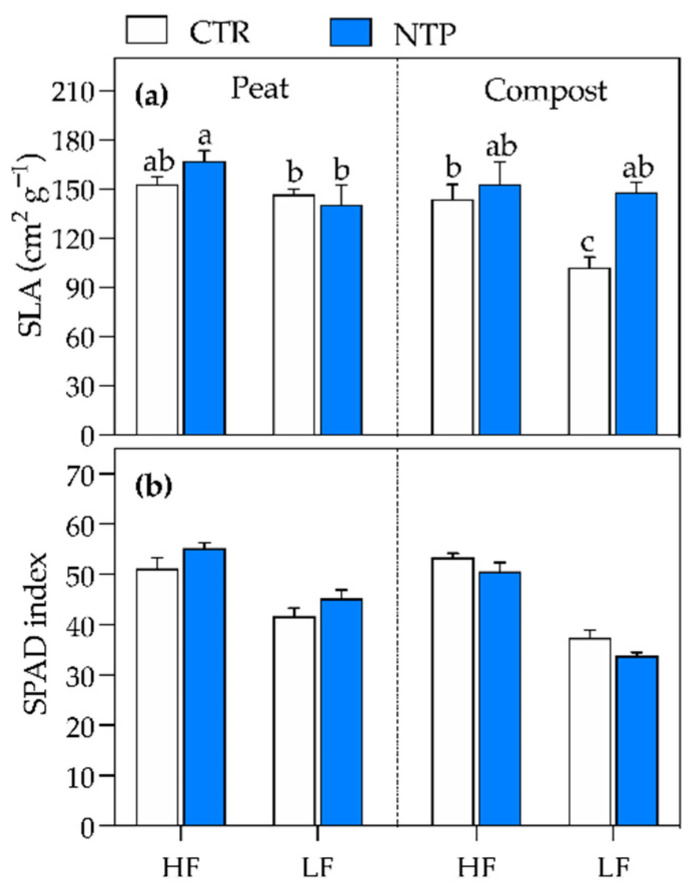
Leaf parameters of gerbera plants grown in peat or compost and fed with high (HF) or low (LF) nutrient solution, untreated (CTR) or NTP-treated (NTP): specific leaf area (SLA) (**a**) and SPAD index (**b**). Values represent the means (*n* = 4) + SD. The presence of different letters represents Tukey’s test results for significant triple interaction, while three-way ANOVA *p*-values are reported in Table 1.

The specific leaf area (SLA) was higher under NTP-HF treatment than in other conditions, while in compost, it was lower for CTR (untreated plants) in combination with LF treatment than in other conditions (Figure 3a, Table 1). Finally, the SPAD index was higher under HF regimes in both peat and compost, while the lowest value was recorded under LF in compost (on average 36 ± 2.2 SPAD units, Figure 3b and Table 1).

### 2.2. Leaf Elemental Concentrations

**Figure 4:**
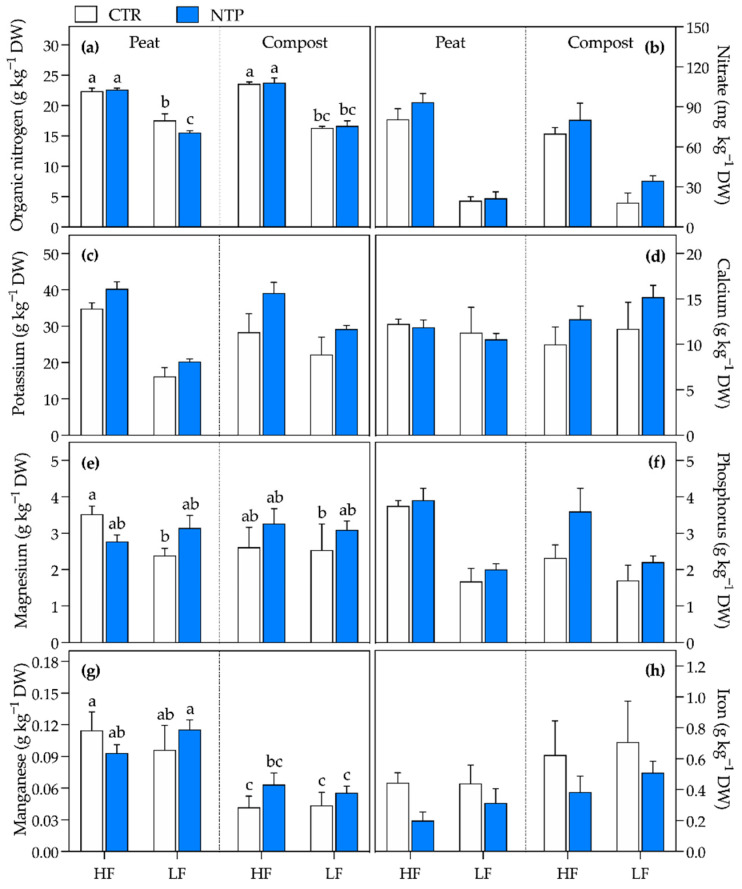
Organic nitrogen (**a**), nitrate (**b**), potassium (**c**), calcium (**d**), magnesium (**e**), phosphorus (**f**), manganese (**g**), and iron (**h**) concentrations within the leaves of gerbera plants grown in peat or compost and fed with a high (HF) or low (LF) nutrient solution, untreated (CTR) or NTP-treated (NTP). Values represent the means (*n* = 4) + SD. The presence of different letters represents Tukey’s test results for significant triple interaction, while three-way ANOVA *p*-values are reported in Table 1.

The combination of substrate, fertilization level, and use of NTP-treated nutrient solution differently affected leaf nutrient concentration (Figure 4, Table 1). The Ca, Mg, Mn, and Fe contents were not influenced by the LF regime, while the other investigated elements all decreased.

Overall, NTP treatment increased the nitric N, K, P, and Mg, while it decreased the Fe concentration. Interestingly, the P, Mg, and Ca concentrations showed a significant interaction between NTP treatment and substrate type, highlighting a higher absorption of these elements when NTP was applied to compost. The substrate influenced organic N, P, Mn, and Fe assimilation and, in particular, the P and Mn contents were found to be higher in peat, while organic N and Fe were higher in compost treatments. Specifically, organic N was higher under a HF regime, without any difference noted between peat and compost. Meanwhile, it was higher in CTR plants than in NTP ones in peat under an LF regime, showing an additional significant interaction between NTP treatment and substrate type. Mn was higher in the peat substrate than in compost. Fe was lower in peat than in compost and decreased under NTP treatment.

### 2.3. Fungi and Bacteria within the Substrate

The quantification of fungi and bacteria allowed the evaluation of the treatment effect on microorganism population levels within the substrates (Table 2). The bacterial and fungal concentrations showed a specular trend: one group increased as the other decreased. The use of compost strongly modulated the fungal presence, decreasing their density, while the level of fertilization and the use of NTP-treated solution did not show any effect on the amounts of fungal colony forming units (CFUs). Overall, the bacterial concentration was higher in compost and, under an LF regime, the use of NTP-treated solution decreased the bacterial CFU in this substrate.

## 3. Discussion

It is well-known that substrate composition may strongly affect the growth and flowering of gerbera plants, presenting a difficulty with peat replacement [19,21,27]. Therefore, agronomic strategies aimed at improving the performance of peat-free substrates are worth investigating. In this experimental trial, compost decreased plant growth and gerbera plants grown in this substrate produced fewer flowers. This effect was probably due to the high pH in the root zone (around 7.0) monitored through percolate analysis (see methodology). Similarly, Sonneveld and Voogt [28] showed an inhibiting effect of high pH on flower number and weight in gerbera plants. High pH in the root zone may induce many detrimental effects on plant physiology; for example, it could induce nutritional impairments in plants and limit the availability of essential nutrients like P, Fe, and Mn [29]. Compost substrate can indeed affect gerbera yield through a decrease in nutrient availability [18], even if it generally contains an extra budget of nutrients [25]. In these experimental conditions, the nutrient concentrations were similar to those reported by other authors [30] but some nutrients were below the optimal concentrations for greenhouse potted gerbera plants, as reported by [31], almost exclusively in plants grown under an LF regime. P and Mn concentrations were significantly decreased in leaves by compost, which would agree with a reduced biomass accumulation, owing to the role that these elements play in photosynthesis [29,32].

Non-thermal plasma treatment is known to decrease the pH of both the substrate and nutrient solution [33], which can be strategic for the maintenance of an optimal pH level in the root zone, especially in those substrates that show a natural tendency to alkalinization. In the present work, pH was adjusted before irrigation so there would be no differences between the untreated and NTP-treated nutrient solutions. The presence of compost caused an alkalinization of the root zone that was not contained by NTP treatment. Yet, NTP may play a role in plant nutrition by adding nutrient elements, like nitric N [2], or by stimulating the release of nutrients (e.g., K), which are potentially available at high concentrations in some composted [25] and uncomposted [26] organic materials. Accordingly, plants supplemented with NTP-treated solution showed an increase in leaf and flower fresh biomasses, but only in peat. The possible role of NTP in growth promotion has already been documented [34]. However, under an LF regime, the NTP treatment had an opposite effect on plant dry biomass, depending on the tested substrate. Particularly, NTP led to an increase in the DW of gerbera plants grown in peat under nutrient shortage, while it worsened the performance of plants grown in compost. Such a DW reduction in compost-grown plants was caused by a significant decrease in % DW, while no significant differences were observed in plant FW. In a preceding study, we reported a decrease in leaf stomatal conductance in lettuce, under NTP treatment [35]. Stomatal activity regulates photosynthesis through CO_2_ uptake and transpiration, controlling water loss; therefore, stomatal closure avoids water loss [36]. A possible reduction in stomatal conductance caused by NTP treatment might increase water reserves, explaining the lack of difference in plant FW. On the other hand, the same reduction in gaseous exchange may have limited CO_2_ intake, thus causing an additional stress, in compost-grown plants. Indeed, stomatal closure is induced by ROS [37,38] that might accumulate under NTP treatment; future research in this regard will help to bring about a better understanding of NTP effects. A slight decrease in % DW was also reported in NTP-treated plants under a HF regime in both peat and compost, but it did not affect plant DW, highlighting a general effect of NTP on this parameter.

Manganese plays a crucial role in photosynthetic activity and antioxidant capacity; the deficiency of this element in plants is commonly caused by alkaline soils [32]. Mn was lower in plants grown in compost than in peat, probably due to a high pH that decreased its availability [39] despite its higher concentration in compost (data not shown). Low leaf Mn concentration in compost might contribute to the lower photosynthetic capacity and biomass allocation found in NTP-LF plants, in combination with the higher SLA, a trait ascribed as an indicator of drought resistance due to the higher photosynthetic capacity of plants carrying this feature [40]. Indeed, under an LF regime, plants grown in peat substrates showed a decrease in SLA, both using untreated and NTP-treated nutrient solution, while plants in compost treated with NTP did not present a reduction in this parameter in comparison with HF regimes. Since NTP promotes antioxidant activity in plants due to ROS production [34], it is plausible that an Mn deficiency can occur at the photosynthetic level in these conditions. This finding would be in agreement with the lowest SPAD units measured under such treatment. Therefore, under a LF regime, plants grown in compost suffered a dual source of stress compared to plants in peat.

Despite the postulated role of NTP as a replacement of N fertilizers [33], the leaf organic N of plants grown in peat under an LF regime was lower in those supplemented with NTP-treated solution than in others. However, nitric N was generally increased by NTP treatment. Indeed, nitrates are the main nitrogen form produced when using NTP to treat water [33]. Moreover, nitrates were higher in percolating nutrient solution of NTP-treated plants than in control ones, under a HF regime, supporting an increase of these molecules following NTP application. NTP treatment decreased the Fe concentration in both substrates, probably influencing its availability. However, plants maintained in compost showed an overall increase in Fe in the aerial part, probably supported by the higher Fe concentration within the compost substrate (data not shown), even if an alkaline condition can decrease Fe availability [41]. On the contrary, NTP increased K, P, and Mg concentrations in plants grown on both substrates.

Nutrient solution optimization to meet actual crop requirements has been reported as a key element to limit nutrient losses from soilless cropping, with low environmental impact [42]. In the present work, the level of fertilization affected both shoot and flower biomass, as well as the low fertilization decreased some nutrients (e.g., N, K, and P), suggesting that an important reduction of those elements is not possible for the correct fertigation of gerbera plants. Nevertheless, the Ca, Mg, Mn, and Fe contents in plants were not affected by fertilization strength, highlighting that a possible reduction of these elements within the nutrient solution allowed fertilizer saving, although further studies are necessary to assess the real minimum concentration thresholds. In the case of peat, NTP treatment increased the leaf and flower biomass of plants grown under nutritional stress, which is worth highlighting.

Although the role of NTP in sanitization has been previously elucidated [4], under these experimental conditions, the use of NTP-treated solution did not show clear antimicrobial effects on fungi and bacteria within any substrate. However, a decrease in bacteria occurred under an LF regime in plants grown in compost, the most stressful situation for gerbera plants, likely due to the potential for this microbial group to be carried into the circulating solution exposed to NTP. Fungal concentration, instead, was lower in compost than in the peat substrate. Compost is an impactful conditioner of the telluric environment that enhances microbial biodiversity, reducing the predominance of one or a few groups on the other, which in other specific cases contributes to the effective suppression of soil-borne fungal pathogens [43]. Under the natural pressure of diseases, no biotic attacks occurred during this trial; therefore, the potential role of NTP treatment in contrasting plant diseases remains to be explored.

In conclusion, NTP treatment promoted the fresh biomass production of gerbera plants grown in peat and nutrient uptake in both substrates, except for Fe, while it decreased the plant dry biomass. Interestingly, NTP had a detrimental effect on the biomasses of plants grown in compost under low fertilization, showing under nutrient shortage a substrate-dependent effect. The penalty in compost could be generally associated with high substrate alkalinization and, under low fertilization, a reduction in photosynthetic capacity, probably further decreased plant growth. The low fertilization treatment highlighted that Ca, Mg, Mn, and Fe could be reduced in respect to the standard fertilization used for gerbera production. The results highlighted as the most recommended treatment a combination of the use of peat along with NTP-treated solution containing a high concentration of nutrients, highlighting the suitability of NTP for horticultural practices. On the other hand, the combination of NTP and compost may not be adopted for this type of ornamental species.

## 4. Materials and Methods

### 4.1. Plant Material, Treatments and Growing Conditions

Seedlings in 104-hole seed trays (around 40 days old) of *Gerbera jamesonii* (L.) ‘Babylon F1′ (Sentier, TV, Italy) were transplanted on 20 February 2019 into 1.5 L pots (∅ 14 cm) in 50:50 peat:perlite (*n* = 320) or 50:50 compost:perlite (*n* = 320). The peat consisted of a mix of white and black Baltic peat with a fine structure (Hawita Professional Spezial Substrate, HAWITA GmbH, Vechta, Germany), while the compost was a green compost provided by Terflor S.R.L. (BS, Italy). The pots were placed on benches (15 plants m^−2^) inside the site-pilot greenhouse described by [44], equipped with NTP technology to treat the nutrient solution. This was located at the CREA Research Center for Vegetable and Ornamental Crops in Pescia, Tuscany, Italy (lat. 43°54′ N, long. 10°42′ E). The NTP was produced by a Dielectric Barrier Discharge device (Jonix SRL, Tribano, PD, Italy) ranging from 5–25 kV, therefore, generating 1012–1015 charged molecules cm^−3^. The nutrient solution was supplied through a drip irrigation system with a flow rate of 2 L h^−1^ pot^−1^, ensuring a leaching fraction of 15–20%, to minimize possible differences between the root zone conditions of plants within the same treatment. For each substrate, plants were randomly divided into eight blocks of 20 plants: four blocks were irrigated with a reference nutrient solution (high fertilization, HF), while the other four blocks were irrigated with a less concentrated nutrient solution (low fertilization, LF). Specifically, the following nutrient solution was supplied as the HF treatment: N-NO_3_ 6.4 mmol L^−1^, N-NH_4_ 0.8 mmol L^−1^, P-PO_4_ 0.8 mmol L^−1^, K 4 mmol L^−1^, Ca 1.8 mmol L^−1^, Mg 0.4 mmol L^−1^, Na 0.31 mmol L^−1^, S-SO_4_ 1.01 mmol L^−1^, Cl 0.26 mmol L^−1^, Fe 16 µmol L^−1^, B 12 µmol L^−1^, Cu 0.4 µmol L^−1^, Zn 2 µmol L^−1^, Mn 2 µmol L^−1^, and Mo 0.4 µmol L^−1^. The nutrient solution supplied as the LF treatment was a quarter of the strength of the standard one. The pH of both nutrient solutions was adjusted to 5.7. Moreover, in a block for each HF and LF of both substrates, the nutrient solution was treated with NTP technology. The nutrient solution was prepared by a fertigation unit and stocked in a tank with a capacity of 1 m^3^ where 0.5 m^3^ of nutrient solution was continuously treated by bubbling NTP-treated air into the solution until a redox potential of roughly 800 mV was achieved.

Eight treatments (*n* = 80, four replicates of 20 plants) were then applied for 11 weeks: 1) standard nutrient solution in peat:perlite substrate (CTR-HF-Peat); 2) NTP-treated standard nutrient solution in peat:perlite substrate (NTP-HF-Peat); 3) low nutrient solution in peat:perlite substrate (CTR-LF-Peat); 4) NTP-treated low nutrient solution in peat:perlite substrate (NTP-LF-Peat); 5) standard nutrient solution in compost:perlite substrate (CTR-HF-Compost); 6) NTP-treated standard nutrient solution in compost:perlite substrate (NTP-HF-Compost); 7) low nutrient solution in compost:perlite substrate (CTR-LF-Compost); 8) NTP-treated low nutrient solution in compost:perlite substrate (NTP-LF-Compost). In summary, three factors of variability were applied with two levels each, i.e., 1) NTP (or not, in the CTR), 2) nutrient solution concentration (standard HF or LF), and 3) substrate (peat or compost, mixed with perlite).

Climate parameters were monitored by meteorological sensors positioned in the cultivation area and recorded on a five-minute basis. The minimum, mean, and maximum daily global radiation were 2.2, 9.8, and 16.2 MJ m^−2^ day^−1^, respectively. The minimum, mean, and maximum daily averages of air temperature were 13.6, 17.7, and 22.1 °C, respectively. The air’s mean daily relative humidity averaged 56.9%.

The percolating nutrient solution was analyzed about every two weeks to monitor the root zone status and the followed parameters were measured: pH, EC, N-NO_3_, and P-PO_4_ (Table 3). N-NO_3_ was measured by the Model Q46F/82 Auto-Chem Fluoride Monitor equipped with a NO_3_ electrode (Analytical Technology, Inc., Collegeville, PA, USA). P-PO_4_ was determined on a nutrient solution using the molybdenum-blue method. Electrical conductivity was two-fold higher in the percolate from a HF regime, in agreement with the higher nutrient concentration (EC of nutrient solution was on average 1400 and 600 µS cm^−2^ for the HF and LF regimes, respectively). Moreover, EC was higher in compost treatments (on average + 250 µS cm^−2^) than in peat. Generally, NTP treatment caused a slight increase in EC, though this was not always observed. The pH in the percolating nutrient solution showed optimal values in peat (on average 5.7) while it was high in compost (on average 7.0), particularly in composted LF plants (+ 0.5 than in composted HF plants). The P-PO_4_ in percolating nutrient solution was two-fold higher in HF treatments than in LF ones without any difference in substrates. On the contrary, N-NO_3_ was higher in peat LF treatment than in compost LF, while the opposite behavior was highlighted under a HF regime.

### 4.2. Plant Sampling and Mineral Element Quantification

The day before plant destructive analysis, SPAD units were measured using a SPAD-502 (Konica Minolta, Chiyoda, Japan) by averaging three measures (basal, median, and apical leaves) on 40 plants per treatment (10 per replicate). On 8 May, 24 plants per treatment (six per replicate) were collected for the final destructive analysis. Therefore, the following measures have been taken as the average value of six plants per replicate. Leaves and flowers were fresh weighted (FW) and, subsequently, oven dried at 65 °C until at a constant dry weight (DW). Specific leaf area (SLA, cm^2^ g^−1^) was also determined as the ratio between plant leaf area, measured through a scanner, and plant leaf DW, using a homogeneous bulk of three leaves from each plant.

Potassium, Ca, Mg, Fe, and Mn were measured by ICP-OES on dried leaf samples (250 mg) following digestion with 5 mL 65% HNO_3_ and 2 mL 85% HClO at 210 °C for 2 h. P was determined on the same digested leaves using the molybdenum-blue method. Organic N was determined on leaves and flowers by Kjeldahl distillation after dry matter digestion with H_2_SO_4_. Nitrates were determined as described by [45], comparing the absorbance at 410 nm against a calibration curve obtained with serial dilutions of a 1000 ppm nitrate standard solution (Merck KGaA, Darmstadt, Germany).

### 4.3. Quantification of Fungi and Bacteria

The abundance of culturable filamentous fungi and total bacteria were evaluated by the serial ten-fold (10^−1^ to 10^−7^) dilution method in three replicates, as reported by [43]. Independent samples for each substrate were used. Fungi were counted on PDA (Oxoid, Thermo Fisher Scientific Inc., Waltham, MA, USA) at pH 6, with 150 mg L^−1^ added of nalidixic acid and 150 mg L^−1^ of streptomycin. Total bacteria were counted on a selective medium (glucose 1 g L^−1^, proteose peptone 3 g L^−1^, yeast extract 1 g L^−1^, K_2_PO_4_ 1 g L^−1^, and agar 15 g L^−1^), with added actidione 100 mg L^−1^. Population densities are reported as the colony forming unit (CFU) g^−1^ DW of the substrate.

### 4.4. Statistics

Data were tested for a normal distribution using the Shapiro–Wilk normality test and were eventually transformed before the ANOVA. Data were analyzed with a three-way ANOVA (NTP, fertilization, and substrate as variables, *p* ≤ 0.05) and later a Tukey’s posthoc test to assess significant differences. Statistical analyses and graphs were performed with Prism 9 (GraphPad Software, Inc., La Jolla, CA, USA).

## Figures and Tables

**Table 1:** The effects of NTP-treatment (NTP), fertilization (F), substrate (S), and their mutual interactions (×) on production, morphometric, and nutrient parameters of gerbera plants (three-way ANOVA results of data reported in Figure 1, Figure 3, and Figure 4).

Parameter	NTP	F	S	NTP × F	NTP × S	F × S	NTP × F × S
Leaf FW	***	***	***	ns	***	***	*
Leaf area	***	***	***	ns	***	***	ns
Flower FW	*	***	***	ns	***	ns	ns
Flower number	ns	***	***	ns	*	**	ns
Plant DW	ns	***	***	*	***	ns	***
Plant % DW	***	***	***	ns	***	ns	***
SLA	***	***	***	ns	***	ns	***
SPAD index	ns	***	***	ns	***	***	ns
Organic nitrogen	ns	***	*	*	*	**	*
Nitrate	***	***	ns	ns	ns	***	ns
Potassium	***	***	ns	ns	ns	***	ns
Calcium	ns	ns	ns	ns	**	*	ns
Magnesium	*	ns	ns	*	*	ns	**
Phosphorus	***	***	**	ns	*	***	ns
Manganese	ns	ns	***	ns	ns	ns	*
Iron	***	ns	***	ns	ns	ns	ns

ns, not significant; * *p* ≤ 0.05; ** *p* ≤ 0.01; *** *p* ≤ 0.001; FW, fresh weight; DW, dry weight; SLA, specific leaf area.

**Table 2:** The effects of NTP treatment (NTP), fertilization (F), substrate (S), and their mutual interactions (×) on the colony-forming units (CFU) of fungi and bacteria within the substrate of gerbera plants grown in peat or compost and fed with high (HF) or low (LF) nutrient solution, untreated (CTR) or NTP-treated (NTP). Values represent the means (*n* = 3) ± SD.

Peat				Compost			
HF		LF		HF		LF	
CTR	NTP	CTR	NTP	CTR	NTP	CTR	NTP
Fungi (CFU × 10^3^ g^−1^)							
0.52 ± 0.032	0.40 ± 0.128	0.57 ± 0.131	0.48 ± 0.057	0.10 ± 0.046	0.06 ± 0.009	0.05 ± 0.029	0.14 ± 0.104
Bacteria (CFU × 10^3^ g^−1^)							
7.6 ± 2.70	17.6 ± 4.51	18.9 ± 11.4	27.0 ± 13.4	32.8 ± 4.86	42.4 ± 13.60	50.8 ± 6.30	29.9 ± 2.70
ANOVA	NTP	F	S	NTP × F	NTP × S	F × S	NTP × F × S
Fungi	ns	ns	***	ns	ns	ns	ns
Bacteria	ns	ns	***	*	ns	ns	ns

ns, not significant; * *p* ≤ 0.05; ** *p* ≤ 0.01; *** *p* ≤ 0.001.

**Table 3:** Chemical parameters of the percolating nutrient solution used for the fertigation of gerbera plants grown in peat or compost and fed with high (HF) or low (LF) nutrient solution, untreated (CTR) or NTP-treated (NTP). Nutrient solutions were sampled every two weeks during the trial and these values represent the means ± SD.

S	F	NTP	EC (µS cm^−2^)	pH	P-PO_4_ (mmol L^−1^)	N-NO_3_ (mmol L^−1^)
Peat	HF	CTR	1114 ± 78.2	5.6 ± 0.14	0.61 ± 0.016	3.8 ± 0.50
		NTP	1305 ± 101.8	5.6 ± 0.14	059 ± 0.010	4.2 ± 0.30
	LF	CTR	582 ± 37.5	6.1 ± 0.17	0.26 ± 0.008	1.4 ± 0.29
		NTP	629 ± 47.6	5.6 ± 0.25	0.26 ± 0.016	1.2 ± 0.33
Compost	HF	CTR	1429 ± 25.4	6.6 ± 0.21	0.61 ± 0.062	4.3 ± 0.54
		NTP	1630 ± 21.3	6.9 ± 0.06	0.58 ± 0.017	4.5 ± 0.64
	LF	CTR	833 ± 57.8	7.4 ± 0.05	0.25 ± 0.018	0.8 ± 0.11
		NTP	752 ± 68.6	7.0 ± 0.44	0.25 ± 0.012	0.8 ± 0.11

## Data Availability

The data presented in this study are available on request from the corresponding author.
